# Verrucous plane xanthomas secondary to lipoprotein X dyslipidemia in the context of cholestatic fulminant hepatitis: A case report

**DOI:** 10.1177/2050313X211057937

**Published:** 2021-11-09

**Authors:** Julie Dang, Darosa Lim, Kevin Watters, Olivier Simard, Karine Doyon, Maxime Rhéaume, Alexandra Mereniuk

**Affiliations:** 1 Division of Dermatology, Department of Medicine, Université de Montréal, Montreal, QC, Canada; 2 Division of Dermatology, Department of Medicine, Hôpital du Sacré-Cœur de Montréal, Montreal, QC, Canada; 3 Department of Pathology, McGill University Health Center, Montreal, QC, Canada; 4 Department of Medical Biochemistry, Hôpital du Sacré-Cœur de Montréal, Montreal, QC, Canada; 5 Division of Hemato-Oncology, Department of Medicine, Hôpital du Sacré-Cœur de Montréal, Montreal, QC, Canada; 6 Division of Internal Medicine, Department of Medicine, Hôpital du Sacré-Cœur de Montréal, Montreal, QC, Canada

**Keywords:** Xanthoma, lipoprotein X, apheresis, dyslipidemia, cholestasis

## Abstract

Cutaneous xanthomas are the result of dermal deposition of lipid, mostly caused by disorders of lipid metabolism. Less commonly, they occur in the setting of cholestatic liver disease, leading to accumulation of lipoprotein X, a rare form of dyslipidemia that does not respond well to conventional treatments. We describe an atypical presentation of sudden diffuse xanthomas secondary to lipoprotein X dyslipidemia in the context of cholestatic fulminant hepatitis caused by trimethoprim-sulfamethoxazole hypersensitivity. Histopathology was also atypical and showed an unusual verrucous appearance consisting of overlying epidermal hyperplasia with hyperkeratosis. Our patient had significant improvement, after normalization of her lipid panel under cholestyramine and 13 sessions of apheresis, with topical corticosteroids offering some relief. This rare case shows the importance of recognizing atypical presentations of xanthomas, particularly when they do not respond to conventional dyslipidemia treatments.

## Introduction

Cutaneous xanthomas are the result of dermal deposition of lipid. They are mostly caused by disorders of lipid metabolism that can be primary or secondary to obesity, diabetes mellitus, cholestatic liver disease and certain medications. The major forms of xanthomas associated with hyperlipidemia are eruptive, tuberous, tendinous and plane (including xanthelasma). Monoclonal gammopathies or lymphoproliferative disorders are other causes of plane xanthomas.

## Case report

A 27-year-old Caucasian woman presented with a 2 week onset of diffuse itchy papules, initially on the eyelids, then on the trunk and extremities. Her medical history included mild atopic dermatitis and 3 month previous fulminant hepatitis with cholestasis and multi-organ involvement secondary to trimethoprim and sulfamethoxazole hypersensitivity. A hepatology consultation and liver biopsy confirmed the diagnosis. For this reason, prior to appearance of the cutaneous lesions, the patient was taking prednisone 7.5 mg PO daily (initially started at 60 mg PO daily 3 months prior) and cholestyramine 12 g PO daily. Family history was negative for dyslipidemia or early cardiovascular events.

**Figure 1.**
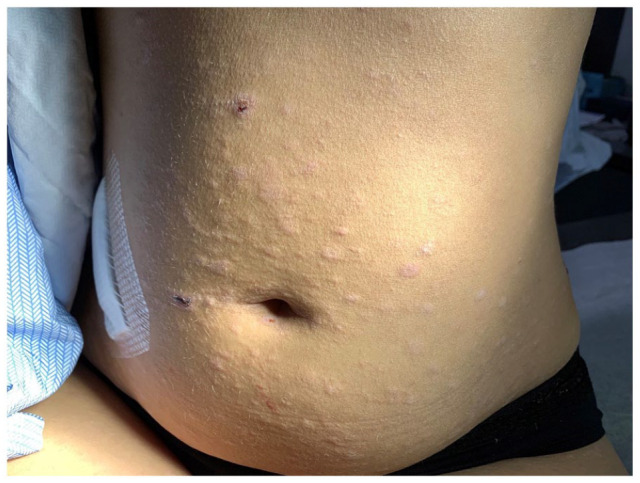
Numerous 1 to 2 cm scaly mildly erythematous papules and plaques on the abdomen.

**Figure 2.**
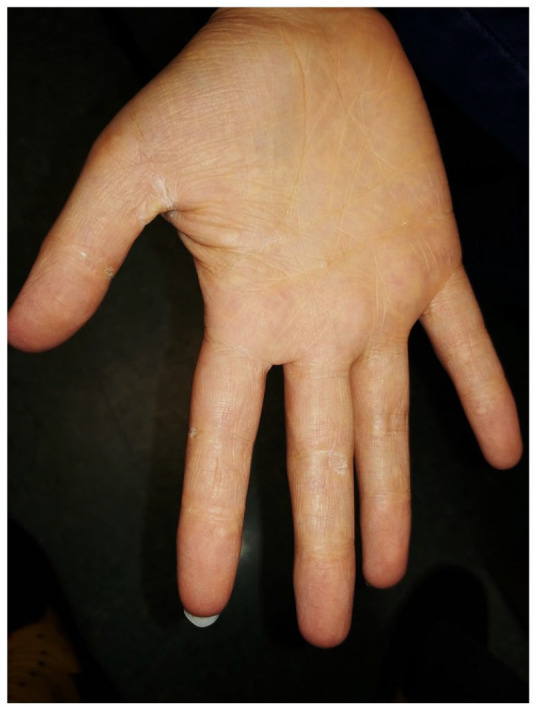
Palmar xerosis and discrete yellowish papules in the palmar creases.

On physical examination, she presented diffuse icterus of sclera and skin as well as numerous 1 to 2 cm scaly mildly erythematous papules and plaques on the abdomen, back, limbs and face, mimicking pityriasis rosea (Figure 1). Furthermore, she had palmar xerosis and discrete yellowish papules in the palmar creases, suggestive of xanthoma striatum palmare (Figure 2).

The patient was concurrently under investigation for hyponatremia; plasma sodium was very low at 123 mmol/L (normal: 134–144) with normal plasma osmolality at 298 mOsm/kg. Workup revealed extremely elevated total cholesterol at 46.84 mmol/L (normal: 3.8–5.2), elevated triglycerides at 5.47 mmol/L (normal: 0.6–2.3), low high-density lipoprotein at 0.19 mmol/L (normal: 0.9–2.2), elevated alanine aminotransferase at 72 U/L (normal: 3.5–50), and elevated conjugated bilirubin at 136 µmol/L (normal: 1.7–8.6), thus confirming a pseudohyponatremia secondary to hypercholesterolemia in the context of hepatic cholestasis. A lipoprotein electrophoresis demonstrated abnormal migration of beta and pre-betalipoproteins in only one band as well as absence of alpha-lipoproteins, which was compatible with lipoproteinemia X dyslipidemia.

**Figure 3.**
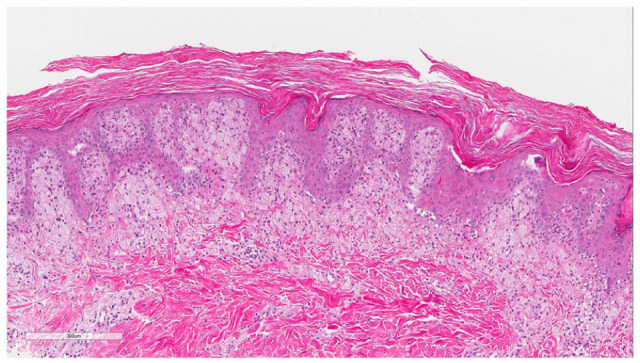
Mild verrucous epidermal hyperplasia with hyperkeratosis and a prominent papillary dermal infiltrate of xanthomatous cells.

A skin biopsy on the flank revealed mild verrucous epidermal hyperplasia with hyperkeratosis and a prominent papillary dermal infiltrate of xanthomatous cells, compatible with verrucous xanthomas (Figure 3).

**Figure 4.**
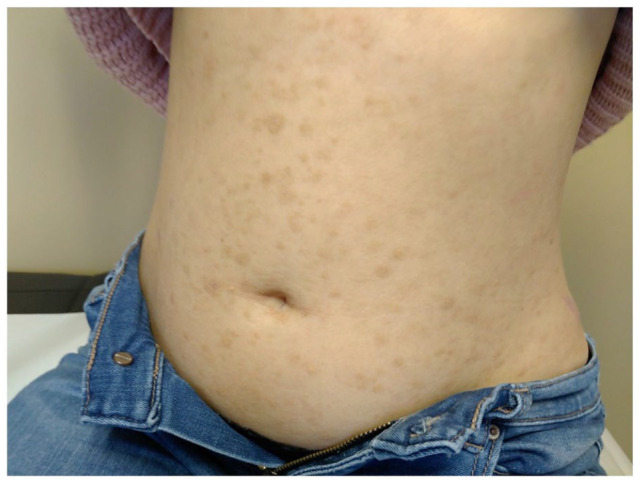
Residual hyperpigmented macules after 10 weeks of apheresis.

A trial of topical corticosteroids prior to confirmation of diagnosis provided mild relief of itch. Given the development of dyslipidemia while already on cholestyramine for 3 months, decision was made to initiate weekly apheresis. As lipid apheresis was not available at our center, conventional apheresis (1 plasma volume exchange per procedure with albumin) was used and still lowered her total cholesterol from 46.84 to 5.74 mmol/L in 10 weeks. It was administered every week for 6 weeks, then every 2 weeks for 6 weeks. After the second procedure, the patient already noticed improvement of the pruritus. After 10 weeks, her lesions improved greatly, leaving residual hyperpigmented macules (Figure 4). To this date, the patient was free of relapse 2 months after discontinuation of apheresis.

## Discussion

We describe an atypical presentation of rapidly occurring diffuse plane xanthomas that were clinically squamous without the classic yellow hue, as well as concomitant xanthoma striatum palmare. In addition, the histopathology presents an unusual verrucous appearance consisting of epidermal hyperplasia with hyperkeratosis, reminiscent of verruciform xanthomas, but with the clinical presentation of plane xanthomas. In fact, verruciform xanthomas are rather solitary plane or verrucous plaques in the mouth or anogenital site. They are not caused by dyslipidemia, but can be associated to lymphedema or epidermolysis bullosa.^1,2^

Another noteworthy feature is that our patient developed these lesions in the setting of lipoproteinemia X secondary to cholestatic hepatitis caused by trimethoprim-sulfamethoxazole hypersensitivity. The diagnosis of hyperlipoproteinemia X was made a few weeks earlier through investigation of sudden-onset hyponatremia with normal plasma osmolality. Lipoprotein X accumulation is a rarely described complication of severe cholestatic liver diseases and is a rare cause of pseudohyponatremia as it interferes with plasma sodium measurements. It is possibly caused by biliary proteins reflux into the systemic circulation.^3^ Therefore, lipid profiles show important elevations of total cholesterol and triglycerides, and low high-density lipoprotein levels.^4^ Because lipoprotein X lacks apoB structure, its accumulation does not respond well to conventional treatments such as statins, fibrates and PCSK9 inhibitors.^5^

A few case reports showed temporary resolution of xanthomas secondary to lipoprotein X with LDL plasmapheresis, but the ultimate treatment remains addressing the underlying condition such as liver transplantation for persisting liver diseases.^3,4,6^ Combination with ursodeoxycholic acid and cholestyramine may also help lower lipoprotein X.^3^ For xanthelasma, destructive methods include surgical excision, cryotherapy, trichloroacetic acid and laser, though the effects are temporary with a risk of scarring.^1,4^ For diffuse plane xanthomas, there are rare case reports of treatments with erbium:YAG laser,^7^ topical simvastatin^8^ and systemic probucol.^9^ We describe a rare case of lipoproteinemia X with complete resolution of xanthomas and normalization of the lipid panel after 18 weeks of conventional apheresis, even without a specific LDL filter.

This rare case highlights the importance of recognizing this atypical clinical presentation and histopathological findings of xanthomas. In the presence of hyperlipidemia, cholestasis and pseudohyponatremia, it is important to perform a lipoprotein electrophoresis to rule out lipoproteinemia X. Investigating possible underlying causes and promptly initiating apheresis can significantly improve the patient’s condition.
